# Prospective evaluation of prognostic factors uPA/PAI-1 in node-negative breast cancer: Phase III NNBC3-Europe trial (AGO, GBG, EORTC-PBG) comparing 6 × FEC versus 3 × FEC/3 × Docetaxel

**DOI:** 10.1186/1471-2407-11-140

**Published:** 2011-04-16

**Authors:** Eva J Kantelhardt, Martina Vetter, Marcus Schmidt, Corinne Veyret, Doris Augustin, Volker Hanf, Christoph Meisner, Daniela Paepke, Manfred Schmitt, Fred Sweep, Gunter von Minckwitz, Pierre-Marie Martin, Fritz Jaenicke, Christoph Thomssen, Nadia Harbeck

**Affiliations:** 1 Klinik und Poliklinik für Gynäkologie, Martin-Luther Universität, Halle Saale, Germany; 2 Klinik für Gynäkologie, Johannes Gutenberg-Universität, Mainz, Germany; 3 Centre Henri Becquerel, OB-GYN, Rouen, France; 4 Klinik für Gynäkologie, Klinikum Deggendorf, Deggendorf, Germany; 5 Klinik für Gynäkologie, Klinikum Fürth, Fürth, Germany; 6 Eberhard Karls Universität, IMB, Tübingen, Germany; 7 Frauenklinik, Technische Universität, München, Germany; 8 Department of Laboratory Medicine Radboud University Nijmegen Medical Cente, Nijmegen, the Netherlands; 9 German Breast Group GmbH, GBG, Neu-Isenburg, Germany; 10 Laboratoire de Transfer en Oncologie Biologieque, l'AP-HM, Marseille, France; 11 Klinik und Poliklinik für Gynäkologie. Universitätsklinikum Hamburg-Eppendorf, Hamburg, Germany; 12 Brustzentrum, Frauenklinik, Universität zu Köln, Cologne, Germany

## Abstract

**Background:**

Today, more than 70% of patients with primary node-negative breast cancer are cured by local therapy alone. Many patients receive overtreatment by adjuvant chemotherapy due to inadequate risk assessment. So far, few clinical trials have prospectively evaluated tumor biology based prognostic factors. Risk assessment by a biological algorithm including invasion factors urokinase-type plasminogen activator (uPA) and its inhibitor plasminogen activator inhibitor type 1 (PAI-1) will assess up to 35-55% of node-negative patients as low-risk and thus avoid chemotherapy. In contrast, a clinical-pathological algorithm will only classify 20-40% of patients as low-risk. High-risk node-negative patients should receive chemotherapy. Anthracycline-based regimens are accepted as a standard, the additional benefit of taxanes remains an open question.

**Methods/Design:**

The international NNBC3 ("Node Negative Breast Cancer 3-Europe") trial compares biological risk assessment (UP) using invasion factors uPA/PAI-1 with a clinical-pathological algorithm (CP). In this trial, the type of risk assessment (CP or UP) was chosen upfront by each center for its patients. Fresh frozen tissue was obtained to determine uPA/PAI-1 using an enzyme-linked immunosorbent assay (ELISA). Patients assessed as high-risk were stratified by human epidermal growth factor receptor 2 (HER2) status and then randomised to receive anthracycline-containing chemotherapy 5-Fluorouracil (F)/Epirubicin (E)/Cyclophosphymide (C) or an anthracycline-taxane sequence (FE_100_C*6 versus FE_100_C*3 followed by Docetaxel_100_*3).

**Discussion:**

In this trial, 4,149 node-negative patients with operable breast cancer from 153 centers in Germany and France were included since 2002. Measurement of uPA/PAI-1 by ELISA was performed with standardised central quality assurance for 2,497 patients (60%) from 56 "UP"-centers. The NNBC 3-Europe trial showed that inclusion of patients into a clinical phase III trial is feasible based on biological testing of fresh frozen tumor material. In addition, 2,661 patients were classified as high-risk and thus received chemotherapy. As adjuvant chemotherapy, 1,334 high-risk patients received FE_100_C-Docetaxel_100_, and 1,327 received French FE_100_C. No unexpected toxicities were observed. Chemotherapy efficacy and comparison of UP with CP will be evaluated after longer follow-up.

**Trial Registration:**

clinical Trials.gov NCT01222052.

## Background

### Breast Cancer in Germany

In Germany, about 58,000 patients are newly diagnosed with breast cancer every year. Today, approximately 80% of patients can expect to be cured or to experience at least long-term survival of more than 10 years. Due to the activities of the national screening program, a growing number of early tumors are detected. Most patients have no or only a few (1-3) axillary lymph nodes involved and therefore have a good chance of being cured. Thus, overtreatment is increasingly becoming an issue.

One of the major clinical questions is how to identify those patients who may be able to avoid adjuvant chemotherapy because of their low risk of recurrence. Better prognostic factors are urgently needed to predict the individual risk of recurrence.

Yet, patients with node-negative disease at high risk of recurrence should receive adjuvant chemotherapy. However, the most effective type of chemotherapy regimen is uncertain. In order to avoid unnecessary side-effects, prospective, randomised controlled comparisons of regimens with and without taxanes are needed.

### Prognostic factors in node-negative breast cancer

#### Clinical and pathological assessment

Most clinicians use grade of differentiation, age, tumor size, steroid hormone receptor status, HER2 expression and sometimes proliferation markers (e.g. Ki-67 or gene signatures like Onco*type*DX™) in order to decide which patient with node-negative disease should receive adjuvant chemotherapy. In case of undifferentiated cancers (grade 3), patients are truly at high-risk and may benefit from chemotherapy, whereas in case of well-differentiated grade 1 cancers, the risk of recurrence may be rather low. However, in the heterogeneous group of grade 2 tumors, it is essential to know for which patients the benefits of chemotherapy will outweigh its potential side effects. The widely used clinical-pathological risk evaluation was defined at the consensus meetings of St. Gallen [1].

#### uPA/PAI-1 for risk evaluation

There is an increasing focus on new biological factors to further assess risk of recurrence in patients with grade 2 breast cancers in order to avoid unnecessary chemotherapy. The "uPA/PAI-1-algorithm" gives promising results [2-4].

The capacity of breast cancer for invasion and early hematogenic metastasis is closely related to the action of receptor-bound, tumor-associated proteases and a central role of the serine protease uPA (urokinase-type plasminogen activator). In independent studies, several groups have shown that the uPA antigen content in tumor tissue is a strong and independent prognostic factor in primary breast cancer [5]. Particularly within the node-negative group, the relative risk of relapse and decreased survival was highest in patients with elevated uPA-levels. In addition, the plasminogen activator inhibitor-type 1 (PAI-1) content in tumour tissue is also related to an increased risk of relapse and decreased survival which becomes visible by PAI-1 being a strong and independent prognostic factor in multivariate analysis. Combining the two invasion factors, uPA and PAI-1, by sequential selection (regression tree analysis) in node-negative breast cancer patients, a high-risk group can be identified comprising about 45% of all node-negative patients. Moreover, the remaining 55% of node-negative patients have an extremely low risk of relapse (93% disease-free survival after 3 years without any adjuvant therapy), so that adjuvant chemotherapy does not seem to be indicated [5,6,2].

In the first prospective, randomized, multicenter trial (Chemo N0), these retrospective data were validated and confirmed [3,7]. In long-term follow-up (10 years), uPA/PAI-1 and tumor grade remained the only independent prognostic factors with a hazard ratio of 3.2 and 2.8, respectively [8].

The Chemo N0 trial demonstrated that determination of uPA and PAI-1 in tumor tissue by ELISA is easily feasible in every laboratory. The international, quality-control system for uPA/PAI-1 determination showed only non-relevant inter-laboratory variations (CV 10-15%) [9]. The clinically most important conclusion from the Chemo N0 data is that at least 44% of all node-negative patients could potentially be spared from adjuvant chemotherapy. Patients with high levels of uPA and/or PAI-1 are at high risk for relapse and should therefore receive optimal adjuvant chemotherapy [10,8]

A pooled analysis by the European Organisation for Research and Treatment of Cancer Receptor and Biomarker Group (EORTC-RBG) used the raw data of most uPA and PAI-1 determinations available at the time worldwide in numerous retrospective and prospective breast cancer studies and confirmed the prognostic value in more than 8,000 patients [2,11,12]. These new prognostic factors, uPA/PAI-1, thus conform to the requirements for clinical acceptance put forward by the late W McGuire and GM Clark in the early nineties [13]. The American Society of Clinical Oncology (ASCO) also added uPA/PAI-1 to the list of recommended prognostic tumor markers for breast cancer [14]. The recommendations for diagnosis and treatment of breast cancer issued by the "Kommission Mamma der Arbeitsgemeinschaft Gynäkologische Onkologie (AGO) e. V. in der Deutschen Gesellschaft für Gynäkologie und Geburtshilfe e. V. sowie in der Deutschen Krebsgesellschaft e. V. " have even supported the use of uPA/PAI-1 in node-negative breast cancer already since 2002 [15].

#### Other biological factors for risk evaluation

The current ASCO guidelines also recommend the use of the 21-Gene assay. This test is available for paraffin embedded tissue. It is based on a reverse transcriptase polymerase chain reaction (RT-PCR) determination of the mRNA expression of 16 tumor-specific genes and 5 control genes. It is widely used in the USA for node-negative or node-positive (1-3 lymph nodes), hormone-receptor positive patients. A high recurrence score is associated with a high probability of recurrence and benefit from adjuvant chemotherapy. This commercially available test (Onko*type*DX™) is currently evaluated in a prospective randomised trial (**T**rial **A**ssigning **I**ndividua**L**ized **O**ptions for Treatment (**Rx**), TAILORx) comparing clinical-pathological versus biological risk assessment by the 21-Gene assay. First results of >10,000 patients are expected at the end of the trial in 2014 [16]. In Germany, the prospective WSG Plan B trial evaluating efficacy of anthracycline-free chemotherapy in primary HER2-negative breast cancer after molecular-based risk assessment according to Oncotype DX and uPA/PAI-1 has already recruited almost 2,500 primary breast cancer patients by early 2011[17].

An additional test, the 70-gene array, is also based on genetic profiling of the tumor. MRNA is prepared from fresh tissue of node-negative and node-positive (1-3 lymph nodes) breast cancer and analyzed by a multi-gene expression-array. This signature is also able to predict an individual patient's risk of recurrence and survival (low vs. high). This commercially available test (MammaPrint™) is currently being evaluated in a prospective randomized trial comparing clinical-pathological versus biological risk assessment. The **M**icroarray **I**n **N**ode-negative and 1 to 3 positive lymph node **D**isease may **A**void **C**hemo**T**herapy (MINDACT) trial started 2006 and will recruit 6,000 patients [18].

Another commercially available gene array to predict the individual risk of recurrence is the 76-gene signature (Rotterdam signature, Affymetrix™) [19]. In addition, the HOXB13:IL17BR ratio index give prognostic information for ER positive tumors and the molecular grade index (five genes) (H/I^SM ^and MGI^SM ^by Biotheranostics™, France) [20,21].

Many promising preclinical results have been published for biomarkers giving not only prognostic information but also predicting therapeutic response and monitoring therapeutic interventions. Information may be derived from the tumor or other patient specimens as mRNA but also DNA, DNA-methylation status, histone markers and miRNA [22].

#### Molecular "intrinsic" typing

Biologically meaningful breast-cancer tumor types have been derived from expression array analyses [23,24]. This molecular typing defines luminal (ER-positive), HER2-type (HER2 overexpressing) and basal-like (often ER and PR and HER2-negative, so called triple-negative) breast cancers, and thus tumor types that respond to specific therapies such as endocrine therapy for luminal and anti-HER2 agents for HER2 type cancers. Luminal breast cancers can be further divided into low-risk (luminal A) and high-risk (luminal B) tumors. Luminal B tumors are highly proliferating [25] and should be treated with chemotherapy in addition to the endocrine therapy. Efforts have been made to substitute gene-expression profiling (requiring frozen tissue) by immunohistochemical analysis of formalin fixed specimen. Since the results of these two techniques do not correlate well so far, their results should still be used with care.

#### Treatment of patients with node-negative breast cancer

In 2005, the Early Breast Cancer Trialists' Collaborative Group (EBCTCG) showed that anthracycline-containing regimens account for up to a 38% (postmenopausal) and 20% (premenopausal) relative reduction in the cumulative 15-year mortality in addition to that attributable to endocrine therapy [26]. Several anthracycline-containing regimens are currently used [15] and so far there is no direct comparison supporting superiority of an individual regimen provided that an adequate dose is used. The EORTC Breast Cancer Group used two different anthracycline-containing regimens as standard in their neo-adjuvant chemotherapy trials. Study 10921 (closed 1996) used Canadian FE120C [27]. Analysis of the dose-intensity data, however, demonstrated that the median dose-intensity delivered for epirubicin was 100 mg/m² for the standard arm because of toxicity leading to early dose adjustment [28]. Consequently, the EORTC trial 10994 (p53 study) used the FE100C regimen as its standard [29] - it also seems to be a feasible standard in the adjuvant setting [30,15].

A recent meta-analysis demonstrated that Taxanes (docetaxel (T), paclitaxel (Pac)) are very active agents in the adjuvant treatment of all breast cancer patient subgroups [31]. There was one major trial particularly comparing a taxane-containing regimen with a solely anthracycline-based regimen in node-negative breast cancer: The GEICAM 9805 trial recruited 1,059 high-risk, node-negative, breast-cancer patients. DocetaxelA50C*6 (TAC) was compared to FA50C*6. The taxane arm was significantly superior regarding disease-free survival (DSF) (hazard ratio (HR) 67%). Overall survival (OS) showed a non-significant trend (HR 0.70) favoring TAC. As expected, side effects were more frequent in the taxane-containing arm [32]. Recently, Docetaxel has also been approved in Europe for treatment of node-negative breast cancer.

The CALGB #9344 trial compared 4 courses of standard AC with a sequence of 4x AC followed by 4 courses of Pac in 3,000 node-positive patients. Patients treated by AC⇒Pac had significantly fewer recurrences [33]. Yet, trial results were discussed controversially: patients in the AC⇒Pac arm were on therapy twice as long as those in the control arm; the beneficial chemotherapy effect may thus be merely attributable to a longer duration of chemotherapy. In addition, retrospective subgroup analyses of this trial showed that the significant advantage of adding paclitaxel was only present in patients with steroid hormone receptor-negative tumors.

Consequently, in the NNBC 3-Europe trial, a balanced comparison between an anthracycline-containing (FE_100_C) and a sequential anthracycline-taxane (FE_100_C-Docetaxel sequence) therapy was chosen with identical dose-intensity and chemotherapy duration in both trial arms. For the taxane-containing sequence, safety data and efficacy data for node-positive disease were already available [34].

### Aim of the NNBC 3-Europe trial

The NNBC 3-Europe trial was aimed to compare biological with clinical-pathological risk assessment in patients with newly-diagnosed node-negative breast cancer. In addition, the question should be answered whether high-risk node-negative patients should receive adjuvant chemotherapy with or without taxanes.

## Methods/Design

### Design of the NNBC 3-Europe trial

#### Overall design

In this international, multicenter, prospectively-randomized, controlled trial, 4,149 node-negative patients with operable breast cancer were included. Risk of recurrence was assessed either by a clinical-pathological (CP) or by a biological algorithm based on uPA/PAI-1 (UP). Type of risk assessment (CP or UP) was decided up-front by each center for all of its patients and then used as a stratification parameter for the trial. Patients assessed as high-risk were stratified by HER2 status and then randomized to receive anthracycline-containing (FE_100_C*6) or anthracycline and taxane-containing (FE_100_C*3 followed by Docetaxel_100_*3) chemotherapy.

A difference regarding disease-free survival between the two chemotherapy arms is expected. Moreover, discordance/concordance between the two methods of prognostic assessment will be compared concerning proportion and DFS of low-risk patients in each of the two risk assessment groups.

The benefit of anthracycline/taxane-containing chemotherapy compared to solely anthracyline-containing chemotherapy will also be evaluated in HER2 positive patients.

Trial objectives:

• Comparison of the chemotherapy regimes

• Comparison of clinical-pathological vs. biological (uPA/PAI-1) risk assessment

• Comparison of the chemotherapy regimes in HER2 over-expressing breast cancer

Ethical approval was obtained by the institutional review board of the principle investigator as well as of each participating center.

### Patients

Patients age 18-65 were eligible for the trial if they had a histological proven primary breast cancer (0.5-5 cm, pN0, M0, R0). For centers using biological risk assessment, frozen tissue had to be available from all patients for uPA/PAI-1 testing. Patients needed to be of adequate health in order to undergo the recommended chemotherapy.

### Procedures

**Figure 1:**
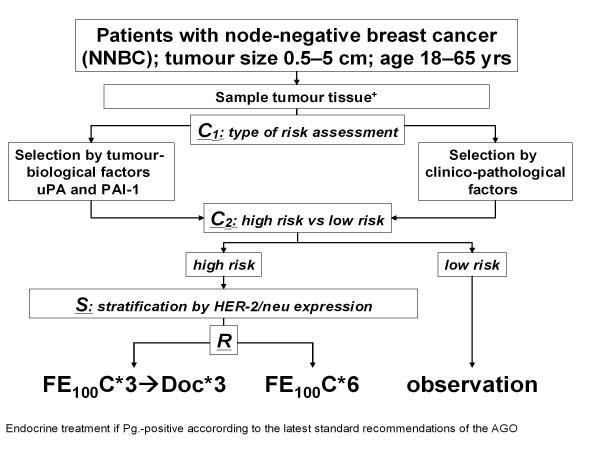
**Design of the NNBC 3-Europe trial**. Stratification and randomization schedule: C1 = stratification by type of risk assessment based on choice of the centre (type of risk assessment); C2 = stratification by risk status; S = Stratification by HER-2/neu-FISH result; R = randomisation (FE100C: 5-Fluorouracil 500 mg/m², Epirubicin 100 mg/m², Cyclophosphamide 500 mg/m², q3 wks; Doc: Docetaxel 100 mg/m², q3 wks.). += fresh tumour tissue for patients within the "biological UP pathway", additionally in all patients paraffin blocks for central review.

After informed consent, patients were registered for the trial (figure 1). In "biological" centers working with UP, frozen tissue was sent to designated laboratories for uPA/PAI-1determination. After confirmation of node-negative disease, patients were classified by their risk status as assessed by either clinical-pathological criteria, or by biological criteria (Classification 1). Patients found to be low-risk according to either CP or UP (see below) were observed; patients found to be high-risk either by CP or UP received adjuvant chemotherapy within the trial (Classification 2). A stratification step was performed according to HER2 status (Stratification). All high-risk patients were randomly assigned to one of the two adjuvant chemotherapy schedules (FEC or FEC-Docetaxel) (Randomization). All patients received breast radiotherapy if breast conserving surgery had been performed. Radiotherapy, endocrine therapy, and trastuzumab were given according to current AGO recommendations at the time (http://www.ago-online.org).

### Risk Assessment

The gold standard to prospectively compare risk assessment procedures would have been randomisation of each patient to either CP or UP risk assessment. To avoid reduced accrual because of physicians' bias or patients not accepting such a rather complicated procedure, consistent risk assessment within each center was chosen as a clinically feasible procedure for the trial. Moreover, not all centers were logistically able to ascertain fresh frozen tissue for UP. Thus, randomization of the centers for method of risk assessment was not feasible. Accounting for individual conditions and preferences, each centre was therefore allowed to select the method of risk assessment for all of their patients.

#### Risk assessment by clinical and pathological factors (CP)

**Figure 2:**
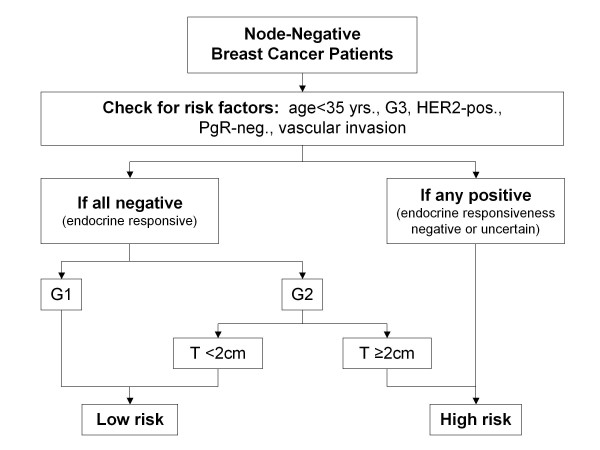
**Risk assessment using the clinical and pathological algorithm (CP) adopted from St. Gallen and NPI**. - all patients G1 or G2 <2 cm were assessed as low-risk if no additional risk factors were present. Patients showing an additional risk factor or G2tumors ≥2 cm or G3 were assessed high-risk.

The integrated clinical-pathological algorithm used to select high-risk patients was derived from the St. Gallen recommendations and the Nottingham Prognostic Index (NPI) system Centers using CP classified all patients younger than 35 years or with a G3 tumor, or with a HER2 positive tumor or with progesterone receptor-negativity or vascular invasion as high-risk. In addition, patients with G2 tumors were considered high-risk if their tumor was ≥2 cm (figure 2).

#### Risk assessment by biological factors (UP)

**Figure 3:**
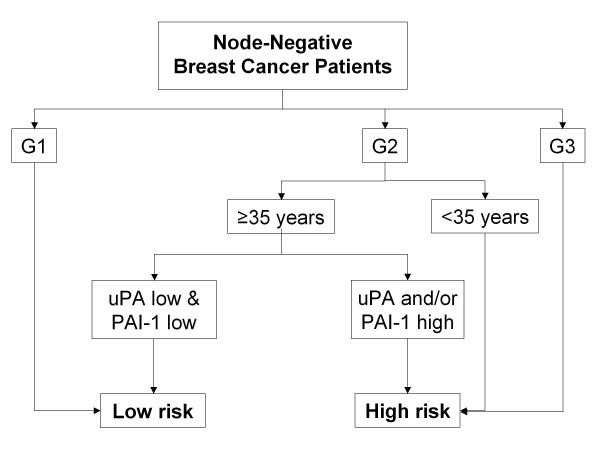
**Risk assessment using the uPA/PAI-1 algorithm (UP) **- all patients with G1 tumors were assessed low-risk, all patients with G3 tumors or ≤35 years were assessed high-risk. Patients with G2 tumors were assessed high-risk if uPA and/or PAI-1 were elevated above the validated cut-off levels.

Centers using biological criteria classified all patients with G3 tumors or <35 years as high-risk, and all patients with G1 tumors as low-risk. Invasion factors uPA/PAI-1 were determined in all G2 tumors. If either factor was above the pre-defined cut-off, the patient was classified as high-risk. The other clinical and pathological factors did not influence biological risk assessment (Figure 3).

### Laboratory procedures

Tissue sampling for biological risk assessment (UP) was done either by excisional or by core-needle biopsy (3 biopsies sent). Core-needle biopsy material has been shown to allow reliable uPA/PAI-1 determination [35]. Regarding excisional biopsies, the pathologist confirmed the diagnosis of breast cancer, excised a representative piece of the tumor (optimum 100-300 mg) and snap-froze it immediately in liquid nitrogen.

The centers received UP results within a maximum duration of 10 days.

In the trial, uPA and PAI-1 concentrations were measured in non-ionic, detergent-released, tumor-tissue extracts (Triton X-100) using the FEMTELLE^® ^ELISA kit #899 by American Diagnostica (cut-off values had been previously validated for this kit [36]). Total protein measurements were performed by the Bicinchoninic acid (BCA) test (Pierce #23225). Tissue handling and methods of measurement have been extensively described elsewhere [37]. Patients with an uPA concentration of ≤3 ng/mg total protein and a PAI-1 concentration of ≤14 ng/mg total protein in the Triton X-100 tissue extract of their primary tumor have a very low risk of relapse and were consequently classified as low-risk [5].

All laboratories participating in this multicenter trial also participated in the EORTC PBG supported international quality assurance program provided by the central trial laboratory (FCGJ Sweep, Nijmegen, Netherlands). The coefficient of variation showed a maximum of 12% for both assays.

### Chemotherapy

All patients received 3 cycles of FE_100_C every 3 weeks consisting of 5-FU 500 mg/m2 IV, epirubicine 100 mg/m2 IV infusion, and cyclophosphamide 500 mg/m^2 ^IV infusion. Prophylactic antiemetic treatment was given according to each center's policy, no prophylactic antibiotics or prophylactic granulocyte-colony stimulating factors (G-CSF) were allowed but secondary prophylaxis was allowed. Patients in the FEC arm then continued for another 3 cycles of FEC. Patients in the docetaxel arm continued with docetaxel 100 mg/m^2 ^IV every 3 weeks. Oral steroids were given for 3 days. Prophylactic G-CSF was recommended according to guidelines. Dose modification was done for hematological or non-hematological toxicity according to the protocol consistent with standard recommendations (National Cancer Institute).

### Radiotherapy, endocrine therapy and trastuzumab

All other adjuvant treatment was given according to current AGO recommendations [38].

### Follow-up

On-site monitoring was performed once for all centers after the last patient was included into the trial. All high-risk and low-risk patients are followed for 5 years. Data is entered by the local centers into an electronic data capture system.

### Statistical analyses

Endpoints were chosen as follows:

1. For the population treated by chemotherapy:

• Primary endpoint of the study is Disease-Free Survival (DFS)

• Secondary endpoints are: Overall Survival (OS) and Safety, and

• side-effects of chemotherapy in each study arm

2. For the entire population of registered patients:

• DFS in each low-risk group (or in each patient group stratified for type of risk selection, CP or UP, respectively), and

• proportion of node-negative breast cancer patients grouped into each low-risk group

### Sample size calculation

#### Questions arising

1. NNBC3-Europe is a randomized, multicenter, open-label, phase III trial designed to detect - with adequate power - a difference in efficacy between two chemotherapy regimens in high-risk node-negative, operable breast cancer.

2. It will also quantify the discordance/accordance of clinical and pathological characteristics and of the biological characteristics with regard to risk assessment in all patients. Therefore, DFS of each low-risk group and of each patient group classified according to type of risk selection, respectively, will be compared as well as the proportion of node-negative breast cancer patients grouped into that particular low-risk group.

3. To investigate whether registered patients (low-risk patients according to their particular risk assessment criterion) can still be discriminated with respect to their risk of first recurrence and survival if the other assessment criterion is used.

4. To investigate prospectively whether patients with HER2 over-expression have a higher benefit by an adjuvant anthracycline-taxane sequence than by a solely anthracycline-based combination.

#### Ad question 1 - difference between chemotherapy regimens

The primary endpoint for the biometrical evaluation is DFS.

Assuming an event rate of 13% at 5 years follow-up in the standard FE_100_C arm, a difference of 4% in the event rate is considered to be clinically relevant.

When the sample size in each group is 1,286, an exponential maximum-likelihood test of equality of survival curves with a 0.050 two-sided significance level will have 80% power to detect the difference between a group 1 exponential parameter of 0.0023 (equivalent to a 13% event rate at 5 years) and a group 2 exponential parameter of 0.0016 (equivalent to a 9% event rate at 5 years) (constant hazard ratio of 1.477).

#### Ad question 2 - difference between risk assessment types

The primary endpoint for the biometrical evaluation is DFS. The power was computed for the question whether the two types of risk assessment are equivalent regarding DSF. The statistical analysis was planned under the condition that patient allocation to one of the two types of risk assessment is not randomized but depends on the policy of each center (see above). Therefore, it was expected that the two groups formed by the risk assessment methods are not homogeneously distributed regarding all relevant prognostic factors.

There is no method described in the literature for an exact power calculation for multivariate statistical methods with event times as the endpoint and equivalence as the main question of the analysis. As an approximation for the power calculation, we used the known calculation for simple proportions as the endpoint and equivalence as the main question. In the final analysis, multivariate methods using DFS as the endpoint will be used.

If sample sizes in the groups are 1,900 and 3,800, a two-group large-sample normal approximation test of proportions with a one-sided 0.010 significance level will have 76% power to reject the null hypothesis that the two groups are not equivalent (the difference in proportions is 0.02 or farther from zero in the same direction) in favor of the alternative hypothesis that the proportions in the two groups are equivalent, assuming that the expected difference in proportions is 0.000 and the overall proportion of disease-free patients is 0.959.

### Closure of recruitment after entering 4,149 patients

In December 2008, the steering committee decided to close recruitment to the trial due to several reasons:

• The estimated number of patients needed to answer the chemotherapy question was definitely reached.

• The answer to the second question concerning the difference between the risk assessment groups will probably be answered since the sample size was calculated rather generously due to lack of an exact power calculation for multivariate statistical methods with event times as the endpoint and equivalence as the main question of the analysis (see above).

• Since initially there was a rather slow inclusion of patients, the actual recruitment fell behind the planned recruitment and premature closure was a predefined possibility.

## Discussion

Estrogen receptor status and HER2 expression are clear predictive factors in breast cancer indicating who will benefit from endocrine and anti-HER2 therapy. No such factor has yet been found to predict response to chemotherapy. So far, only some clinical and pathological factors estimating prognosis have been identified. Since chemotherapy is associated with side-effects and reduced quality of life, the indications should be carefully decided. Node-positive patients will most likely benefit from adjuvant chemotherapy in a significant way. Node-negative patients are a heterogeneous group where 70% of the patients will be cured by loco-regional therapy alone. Especially for patients with G2-tumours, there are no good prognostic factors. Recently, numerous biological tests have been suggested for risk estimation in such cases. Only a few are currently recommended by international guidelines (AGO, ASCO, National Comprehensive Cancer Network). uPA/PAI-1 have been evaluated by a prospective trial showing independent prognostic value even after 10 years of follow-up (Chemo N0 trial). A meta-analysis including more than 8,000 patients validated this independent prognostic value. This NNBC-3 Europe trial is the confirmatory trial regarding the prognostic value of these factors. Moreover, the important questions of optimal chemotherapy in high-risk node-negative patients and of identifying node-negative patients benefitting from adjuvant taxane-containing therapy are addressed. 4,149 patients were included and the recruitment was closed in January 2009. First results will be available in 2011 when 142 events have been observed.

## Competing interests

The trial has received unrestricted funding for the trial from Sanofi-Aventis Deutschland GmbH Pfizer Pharma GmbH and Roche Pharma AG. The trial was supported by NBL Program Martin-Luther-University # FKZ 15/29. It is possible that these organizations may gain or loose financially from the publication of this manuscript. The authors declare that they have no other competing interests.

## Authors' contributions

EJK made substantial contributions to the concept and design of the study, coordinates the study and has drafted the manuscript; MV made substantial contributions to the concept and design of the study, coordinates laboratory testing and has helped drafting the manuscript; MS made substantial contributions to the concept and design of the study, coordinates the study and critically revised the manuscript; CV coordinates the study and critically revised the manuscript; DA coordinates the study and critically revised the manuscript; VH coordinates the study and critically revised the manuscript; CM made substantial contributions to the concept and design of the statistical part of the study, coordinates the statistics of the study and critically revised the manuscript; PD coordinates the study and critically revised the manuscript; MS made substantial contributions to the concept and design of the study, coordinates laboratory testing and critically revised the manuscript; FS made substantial contributions to the concept and design of the trial, coordinates laboratory testing and critically revised the manuscript; GvM made substantial contributions to the concept and design of the trial, coordinates the trial and critically revised the manuscript; PM made substantial contributions to the concept and design of the trial, coordinates the trial in France and critically revised the manuscript; FJ made substantial contributions to the concept and design of the trial, coordinates the trial and critically revised the manuscript; CT is responsible for concept and design of the trial, is principal investigator of the trial and critically revised the manuscript; NH is responsible for concept and design of the trial, is principal investigator of the trial and critically revised the manuscript.

All authors read and approved the final manuscript.

## Pre-publication history

The pre-publication history for this paper can be accessed here:

http://www.biomedcentral.com/1471-2407/11/140/prepub
